# Deterministic and Stochastic Processes Regulate Co‐Occurrence Network Structure and Shape Macroinvertebrate Diversity in Karst Environment

**DOI:** 10.1002/ece3.72802

**Published:** 2026-01-06

**Authors:** Wei Liu, Mengzhen Xu, Giri R. Kattel, Xudong Fu

**Affiliations:** ^1^ State Key Laboratory of Hydroscience and Engineering Tsinghua University Beijing China; ^2^ Department of Infrastructure Engineering The University of Melbourne Parkville Victoria Australia

**Keywords:** cave‐reservoir‐and‐stream ecotone, co‐occurrence network, deterministic and stochastic processes, karst landscape, macroinvertebrate community, Southwest China

## Abstract

Biodiversity and ecosystems around the world are consistently threatened by different factors. How deterministic processes and stochastic processes shape biodiversity have become one of the central questions in ecological studies today. The karst region of Southwest China's Yunnan Province is one of the world's biodiversity hotspots and features unique surface and underground riverine landscapes. We studied macroinvertebrate communities in a Cave‐Reservoir‐and‐Stream (CRS) ecotone to assess drivers of macroinvertebrate diversity in the region. Our results demonstrated that in co‐occurrence network, keystone taxa such as *Trigomphus* (betweenness = 0.303, degree = 0.298) contributed to shape the community assembly and maintain diversity, while modules (subcommunities of closely related taxa) promoted species coexistence and interactions further enhancing taxonomic and functional diversity. The diameter and average path length (APL) of co‐occurrence network of cave, reservoir, and stream were 2.83, 3.98, 8.10 and 1.27, 1.43, 2.82, respectively, with 17.4%, 12.0%, and 5.0% isolated taxa and 5, 4, and 9 modules. Stochastic processes would maintain diversity and community assembly when the environment favored taxa growth, while deterministic processes would become critical under deteriorated environmental condition due to the loss of keystone taxa. Deterministic processes dominate in caves (73.6%), stochastic processes in streams (85.9%), while in lakes the two processes are almost similar. This study provided insights into the significance of riverine connectivity in the karst landscape ecotone for maintenance and conservation of endemic freshwater ecosystem and diversity and contributing to important ecosystem services in southwest China.

## Graphical abstract

Keystone taxa and co‐occurrence network modules are fundamental basis for maintaining biodiversity in macroinvertebrate communities. Modules enhance interactions among macroinvertebrates and promote species coexistence which further influences diversity. Stochastic processes play a stronger role in the maintenance of diversity than deterministic processes under suitable environmental conditions, however deterministic processes become the main driving force that leads to keystone taxa loss and modules disconnected in degraded environments.

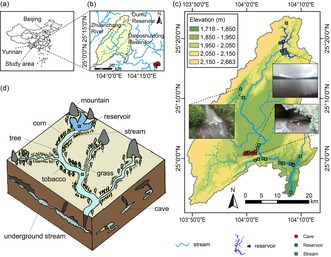

## 1 Introduction

Ecosystems and biodiversity have faced unprecedented challenges of conservation worldwide. Freshwater ecosystems representing 0.01% global water resource covering about 0.8% area, support 6% of all described species worldwide (Dudgeon et al. 2006). However, freshwater ecosystems are threatened by anthropogenic disturbances, climate change, water shortages and pollution. By early this century, more than 65% global river discharge declined due to population growth and climate warming leading to habitat loss and species extinction (Vörösmarty et al. 2010). Advancement in agriculture and the rapid use of renewable energy through the construction of irrigation and hydropower dams in the world's river basins have further disrupted river flows and water quality causing water stresses among both people and nature (Kattel 2019). The ecologically healthy river basins around the world are largely dependent on how anthropogenic forces and natural climate variability during the 21st century are balanced with the use of advanced knowledge and technology in ecosystem functioning and biodiversity conservation and management (Vörösmarty et al. 2010; Kattel et al. 2021).

Karst landscapes around the world feature some of the most significant three‐dimensional ecotones, that include groundwater, surface water, and terrestrial ecosystems. The three prominent ecosystems, covering more than 15% of the world's ice free terrestrial surface area, interplaying very complex bio‐physical interactions within the karst landscapes (Zhao et al. 2011). The continental Asia features the largest karst area extending as high as 8.35 million km^2^ (Goldscheider et al. 2020). The watersheds of the Asia's vast karst landscapes are the homes of 700 million people (Goldscheider et al. 2020), and often regarded as the “natural laboratories” (Carroll and Thorp 2014). With complex ecosystems and rich biological diversity over the millennia, the karst‐dominant watersheds in Asia have been the few remaining places in Earth, being able to protect cosmopolitan, rare, and unique floral and faunal species (Barquín and Scarsbrook 2008). For instance, the China's Yunnan‐Guizhou Plateau is one of the largest karst areas in Asia, has protected the unique set of troglobitic and cavefish species that are also regarded as vulnerable species in the world (Zhao et al. 2011). However, lately the Yunnan‐Guizhou cave ecosystems in southwest China are threatened by a range of environmental stressors including water pollution, pumping, soil erosion, overfishing, mining, and climate change such as prolonged droughts and episodic floods (Reid et al. 2012). These pressures have severely impacting the endemic aquatic species diversity, and some of them are reportedly becoming extinct (Shu et al. 2013).

Comprehending the mechanisms behind the drivers of ecosystem and biodiversity change in Southwest China's cave ecotone is essential to inform better ecosystem management and conservation in the future. Understanding anthropogenic activities, and how they shape endemic karst ecosystem and biodiversity in southwest China is significant for formulating effective strategies for sustainable ecosystem management in the region (Liang et al. 2025). Outlining how species diversity and their contributions to ecosystem services in the karst environment is crucial for devising long‐term goals in conservation policies in the region (Dee et al. 2019). However, although some hypotheses are being tested, what determines the biodiversity, dynamics, and change, is not yet sufficiently understood among ecologists around the world (Chase 2010; Zhou et al. 2013; Schmera et al. 2017). As taxonomic diversity does not capture the differences among individual species groups, functional diversity plays a significant role in distinguishing how functional groups of species would influence ecosystem functioning (Schmera et al. 2017). In the context of southwest China's karst environment, functional diversity of biota is rarely investigated, and any test of hypothesis related to functional diversity and ecosystems is not available.

For understanding diversity, two key sets of processes, deterministic processes and stochastic processes, need to be well established (Zhou et al. 2013). Deterministic processes involve complex interactions between biota and their environments including both abiotic and biotic components. Certain environmental factors can be deterministic to shape macroinvertebrate communities and diversity, such as streambed sediment, suspended fine sediment, stream current power etc. (Duan et al. 2009; Zhou et al. 2017; McKenzie et al. 2020). However, in stochastic processes, an ecological drift may occur, which leads to changes in the species assembly, distribution, and succession in the ecosystem (Chase 2010). Such changes are often mediated by probabilistic events such as dispersal, birth, death, and immigration (Dini‐Andreote et al. 2015; Liu et al. 2024). Moreover, keystone taxa maintain structure and diversity, any drastic loss of keystone taxa may lead to a cascading effect on ecosystem and species assembly (Banerjee et al. 2018). However, the understanding of deterministic processes and stochastic processes and their implications for beta diversity is often hampered by not only the complex and multi‐dimensional ecosystems (Milner and Robertson 2010) but also the mechanisms of these processes. Such rapidly dynamic processes and assembly changes in karst ecosystems are largely unknown. A Cave‐Reservoir‐and‐Stream (CRS) ecotone in Southwest China, which contains rich macroinvertebrate communities is selected for assessing how deterministic processes and stochastic processes are shaping macroinvertebrate diversity. Hence, establishing compositional differences of macroinvertebrates across the CRS ecotone holds considerable significance in establishing the dynamics of species diversity in southwest China's karst landscapes. In such ecotone, co‐occurrence relationships among biota play a fundamental role in forming ecological patterns, and maintain diversity (Williams et al. 2014). The co‐occurrence network analysis is valuable for identifying keystone taxa and delineating subcommunities within macroinvertebrate communities, thereby yield holistic views of ecological patterns across different landscapes, where the CRS ecotone is no exception (Banerjee et al. 2016, 2018; Pilosof et al. 2020). In this study, the co‐occurrence network analysis was applied to distinguish the keystone taxa (important taxa within communities) and subcommunities (referred to as modules within the co‐occurrence network) in maintaining macroinvertebrate community structure and diversity.

Our specific aims were therefore to: (1) evaluate habitat heterogeneity, taxonomic diversity, functional diversity and β diversity of macroinvertebrates in the CRS ecotone; (2) identify fundamental units—specifically keystone taxa and subcommunities—that maintain macroinvertebrate diversity; and (3) examine how deterministic and stochastic processes shape diversity and evaluate their contributions to determining the state of biodiversity. We proposed a dual‐process analysis framework: firstly, decompose roles of deterministic processes through co‐occurrence networks of the CRS ecotone, with emphasis on subcommunities and keystone taxa; secondly, used the null model provide a potential to reveal the role of stochastic processes. The results will benefit biodiversity conservation and management in karst environments globally.

## 2 Methods

### 2.1 Study Area

**FIGURE 1:**
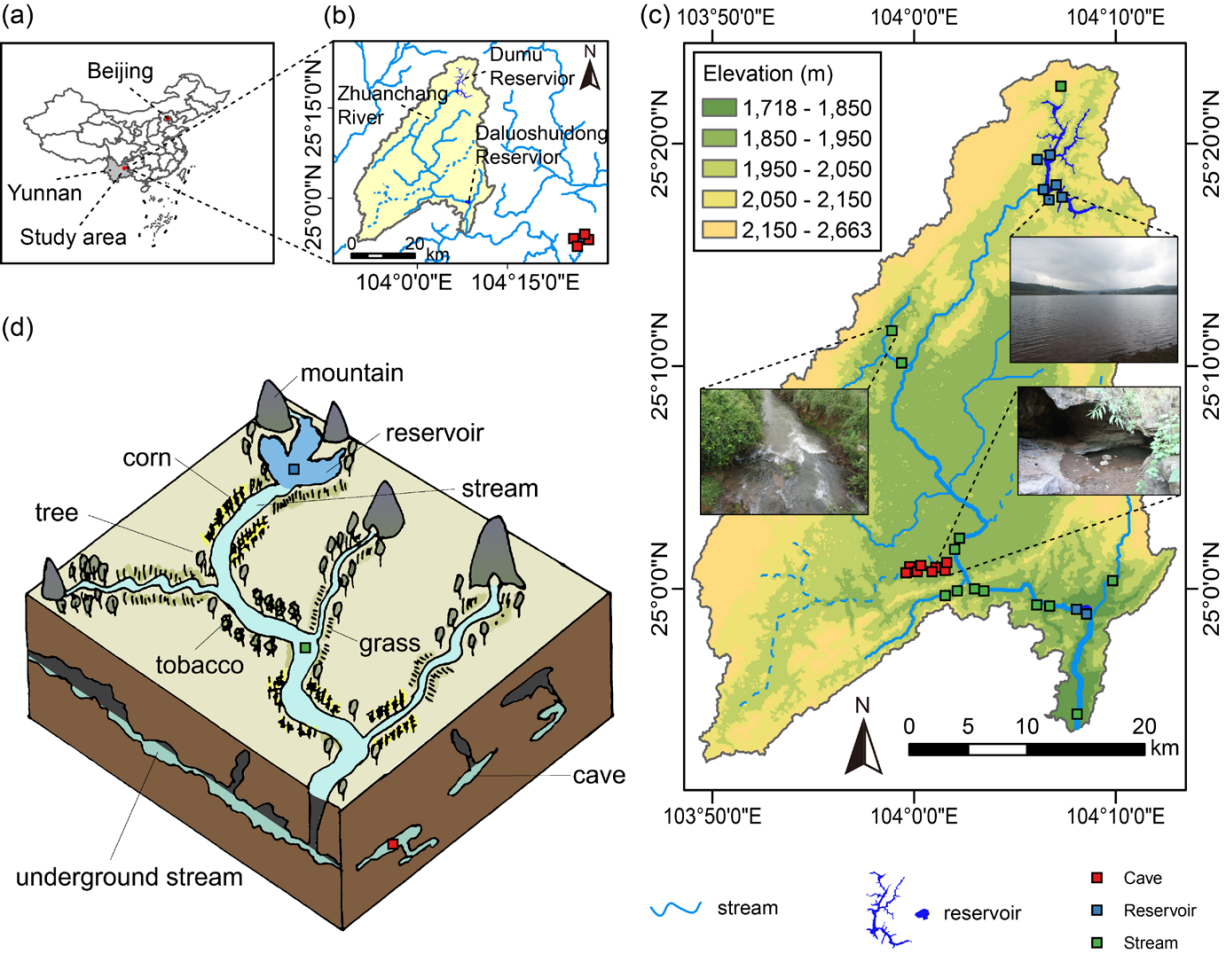
Study area. (a) Yunnan Province, Southwest China; (b) the Zhuanchanghe River basin, the four Cave sampling sites (the red dots) were located outside of the Zhuanchanghe River basin from ground surface but actually connected to it through underground rivers; (c) the sampling sites in the CRS ecotone, the red, blue, and green dots indicate the sampling sites of caves, reservoirs, and streams, respectively; (d) Schematic example of the characteristic network of the Cave‐Reservoir‐and‐Stream (CRS) ecotone.

The CRS ecotone was selected in the Zhuanchanghe River basin, which belongs to the upper Pearl River system, located in Yunnan Province, Southwest China. The Zhuanchanghe River originates from the Dumu Reservoir and flows through the Daluoshuidong Reservoir, then ultimately joins the Jiulong River, stretching approximately 70 km in total length. The basin exhibits an elevation range of 1531 to 2087 m, covers a catchment area of 196 km^2^, receives an annual precipitation of 1100–1400 mm and the cumulative runoff in a year of the catchment is 654.9 million m^3^. Yunnan Province contains the world‐renowned karst landscapes and is a global hotspot for biodiversity (Shu et al. 2013). The Zhuanchanghe River is representative of the karst river systems in the province, with a small number of tributaries and a vast, connected groundwater river network (the study area and the schematic diagram of CRS ecotone see Figure 1). The selected CRS ecotone allows survey of macroinvertebrate communities within the same species bank under different aquatic habitats of karst environment: underground cave‐dominated freshwater system (Cave), standing water dominated by reservoirs (Reservoir) and flowing water dominated by streams (Stream). Twelve sites were sampled once in Cave, 8 sites in Reservoir, and 13 sites in Stream habitats from January 2016 to May 2017 (Figure 1b,c).

### 2.2 Macroinvertebrate Sampling and Environmental Variable Measurement

Three subsamples of macroinvertebrates were collected at each sampling site in the profundal, transition and vegetated littoral zones, for evaluating assemblage diversity and composition. Each subsample was collected from an area of about 1/3 m^2^, using a kick‐net with a cross‐sectional area of 1 m^2^ and a mesh size of 420 μm. Three subsamples from one sample site were mixed into a single composite sample. Specimens were manually picked and preserved in 75% ethanol.

During macroinvertebrate sampling, environmental variables were measured. For each sampling site, coordinates and altitude were measured using an iHand differential GPS tool (GPS 72H, China). Flow velocity (FV) was measured using a propeller‐type current meter (Model LS 1206B, China). After collection, substrate samples were dried and manually sorted using standard sieving and the 50th percentile particulate size (D50) was determined and used to represent the average particle size of substrates (Zhou et al. 2019). A digital lux meter (TA8120, China) was used to measure light intensity (LI). EXO Sondes and EXO Handheld System (Xylem, USA) were used to determine water quality parameters in situ, including water temperature (WT), dissolved oxygen (DO), pH (pH), conductivity (Cond), and concentration of chlorophyll a (CA). In addition, 500 mL water samples were taken (250 mL from near the surface and 250 mL from near the stream/reservoir bed) for nutrient concentration analyses. Total nitrogen (TN) and total phosphorus (TP) concentration were determined in laboratory following Standard Methods for Water and Wastewater Monitoring and Analysis (Liu et al. 2019).

### 2.3 Identification, Measurement and Diversity Indices of Macroinvertebrate

Most specimens were classified at the genus level, while others were divided into distinct morphological types under dissecting microscope (XYH‐3A, China) and optical microscopes (XSP‐8CA, China) (Morse et al. 1994; Epler 2001; Wiggins 2015). The density of individuals was counted as ind.·m^−2^, and the wet weight was determined to the nearest 0.1 mg using an analytical balance. Specimens were categorized into eight functional feeding groups (FFGs): filter‐collector (FC), gatherer‐collector (GC), omnivore (OM), parasite (PA), piercer (PI), predator (PR), scraper (SC), and shredder (SH) (Barbour et al. 1999). Taxa with multiple possible feeding strategies were assigned to a single FFG following its predominant feeding behavior (Palmer et al. 1996).

One taxonomic diversity index (the taxa richness) and one functional diversity index (the FFG richness) of macroinvertebrate communities were calculated based on taxon abundance to assess α diversities. One β diversity index was calculated based on a series of pairwise Bray–Curtis dissimilarity metrics between each two sampling sites in a group. For example, the pairwise dissimilarity metrics of sample Cave site A and sample Cave site B were computed with the “vegdist” function of the “vegan” package and calculated with Equation (1),

(1) $${\mathrm{BC}}_{\mathrm{jk}}=\frac{\sum |y_{\mathrm{ij}}-y_{\mathrm{ik}}|}{\sum \left(y_{\mathrm{ij}}+y_{\mathrm{ik}}\right)}$$

where *BC*_*jk*_ is the Bray–Curtis dissimilarity between sample *j* and sample *k*, $y_{\mathrm{ij}}$ is the abundance of taxa *i* occurred in sample *j*, while $y_{\mathrm{ik}}$ is the total abundance of taxa *i* occurred in *j*. Taxa dominance was calculated with Equation (2) (Liu et al. 2019),

(2) $$\text{Dominance}=\frac{n_{i}}{N}f_{i}$$

where *n*_*i*_ is the abundance of the *i*‐th taxa in each sampling site, *N* is the total number of macroinvertebrate individuals, and *f*_*i*_ is the frequency of occurrence of the *i*‐th taxa in the sampling site.

### 2.4 Co‐Occurrence Networks Analysis

The co‐occurrence networks of Cave, Reservoir and Stream were constructed based on adjacency matrices derived from taxa abundance matrices within the CRS ecotone, using the “igraph” package (de Vries et al. 2018). The “psych” package was used to calculate adjacency matrices and the significant (*p* < 0.05, |r| > 0.6) correlation matrices were selected. The “ggraph” package was used to visualize them. The network nodes were the taxa in the community, an edge between pairs of nodes indicated a potential interaction between the corresponding taxa. The isolated nodes were the taxa that were not involved in the networks, and their ratio was calculated relative to the total number of nodes.

Degree and betweenness were the two commonly used centrality indices for evaluating nodes (McClure 2019; de Vries et al. 2018; Shi et al. 2016). Degree of a node refers to its total number of links to other nodes, and betweenness measures how frequently a node lies on the shortest path between two other nodes. To facilitate the comparison of nodes in networks with different sizes, normalized degree and betweenness were calculated with Equations (3) and (4) respectively:

(3) $$\text{Degree}=\frac{D_{\left(v\right)}}{n-1}$$

(4) $$\text{Betweenness}=\frac{2}{\left(n-1\right)\left(n-2\right)}\sum_{s\neq v\neq t}\frac{\sigma_{\mathrm{st}}\left(v\right)}{\sigma_{\mathrm{st}}}$$

where n denotes the total number of nodes in the network, represent the number of taxa in the community; $D_{\left(v\right)}$ is the number of directly connected neighboring nodes to node v, represent potential interactions betweenness one taxon and other taxa; $\sigma_{\mathrm{st}}$ is the total number of the shortest paths between nodes s and t; and the $\sigma_{\mathrm{st}}\left(v\right)$ indicates the number of these shortest paths that traverse node v. Therefore, higher degrees of the node taxa indicate that they have potential interactions with more other taxa, and higher betweennesses show that more taxa associated with each other through the node taxa (Toju et al. 2017). The degrees and betweennesses were computed with the “degree” and “betweenness” functions of the “igraph” package.

The modules represent highly connected node structures within the networks, reflecting the sub‐community structure of the macroinvertebrate assemblage (Pilosof et al. 2020). The modules of the networks were identified using the Girvan‐Newman algorithm, a method for detecting community structures based on edge betweenness (Newman and Girvan 2004). The central premise of this algorithm is that edges connecting different communities exhibit higher betweenness than those within a module, as they are traversed by a greater number of shortest paths connecting nodes from distinct subcommunities. The algorithm successively removes the edge with the highest betweenness and recalculates the betweenness for all remaining edges after each removal. The entire process of edge removal is recorded. After all edges are removed, the modularity (a measure for choosing the number of modules) is computed for the entire sequence of partitions generated during the process. The optimal community partition is determined by selecting the partition at which the modularity value reaches its maximum, thereby reflecting the highest quality of division. This detection was performed using the “cluster_edge_betweenness” function of the “igraph” package.

Through different interim nodes and different edges, there could be different paths between two nodes. The length of a path was defined by its number of edges. Distance between two nodes was represented by the minimum length among all the paths. The longest distance in a network or a module was regarded as diameter, and the mean distance was regarded as average path length (APL), calculated with Equations (5) and (6):

(5) $$\text{Diameter}=\max\left\{d\left(s,t\right)\;|\;s,t\in V,s\neq t\right\}$$

(6) $$\mathrm{APL}=\frac{2}{n\left(n-1\right)}\sum_{1\leq s<t\leq n}d\left(s,t\right)$$

where V denote the set of all network nodes, $d\left(s,t\right)$ represents the shortest path length between nodes s and t, and n is the total number of nodes in the network. When calculating the diameters and APLs of the network, if the network is disconnected, the analysis shall be restricted to its largest connected component. Lower APL indicates that more pairs of nodes may have a directly linkage (directly interaction), while higher APL showed more indirect linkages or a long path including many nodes. The diameters and APLs were computed with the “diameter” and “average.path.length” functions of the “igraph” package.

In this study, keystone taxa are defined as those exhibiting the highest values of degree or betweenness centrality (Banerjee et al. 2016; McClure 2019). The implications of their loss on community structure are assessed by simulating their removal and examining the resulting changes in network topology. To investigate potential consequences of taxonomic loss, taxa were sequentially removed one by one according to one of four removal sequences: descending order of degree centrality, descending order of betweenness centrality, descending order of dominance, and random order. For Cave, Reservoir, and Stream, random removal sequences were generated by random sampling across each habitat type, with 15 independent replicates per habitat. This procedure was employed to generate new co‐occurrence networks. The diameters and APLs of these modified networks were compared to those of the original full co‐occurrence networks of the CRS ecotone, to assess the effects of losing keystone taxa, dominance taxa, and randomly selected taxa.

### 2.5 Macroinvertebrate‐Environment Relationships Analysis

The comprehensive datasets of Cave, Reservoir, and Stream macroinvertebrate taxa and environmental parameters were used in the analysis of macroinvertebrate‐environment relationships of the CRS ecotone. Detrended correspondence analysis (DCA) was first conducted to choose a model for constrained ordination. If the value of the longest gradient length of DCA exceeded 4.0, a unimodal response model such as canonical correspondence analysis (CCA) was adopted; otherwise, a linear model such as (RDA) was applied (Lepš and Šmilauer 2003). Subsequently, CCA was performed to explore the relationships between macroinvertebrate and their environmental parameters. Prior to conducting CCA, we excluded environmental factors that show statistically significant correlations to avoid multicollinearity. To assess this, Pearson correlation was used to evaluate the relationships among environmental factors. DCA and CCA performed using the “vegan” package, while relationships among environmental factors were assessed using the “PerformanceAnalytics” package.

To reveal the role of stochastic processes in the macroinvertebrate‐environment relationships of the CRS ecotone, 66 null model communities were compared to the real communities to distinguish Bray–Curtis dissimilarity between them (Chase 2010). These null model communities were constructed by randomly sampling from all macroinvertebrate documented in the CRS ecotone for this study. To ensure that rare taxa in real communities remained rare in the null model communities, we preserved the occurrence frequencies of all taxa throughout the randomization process. The higher the similarity in the macroinvertebrate composition and structure between the stochastic communities and the real one the more likely that stochastic processes played an important role in the CRS ecotone. The “randomizeMatrix” function and the “frequency” method from the “picante” package in R were used to randomize species composition and generate community abundance matrices. Meanwhile, the normalized stochasticity ratio (NST) method was employed to quantify the contributions of stochastic processes and deterministic processes (Ning et al. 2019), using the “tNST” function and “bray” method from the “NST” package.

### 2.6 Statistical Analyses

Analysis of variance (ANOVA) was conducted to evaluate significant differences in each environmental parameter and diversity index across the CRS ecotone, with post hoc Tukey HSD tests conducted to identify significant differences among Cave, Reservoir, and Stream. The analysis was performed using the “stats” package. Kruskal‐Wallis rank sum test and multiple comparison test after Kruskal‐Wallis test were used to show the overall significant differences and the differences between two levels of taxa dominance or two ecotones, using the “stats” package and the “pgirmess” package accordingly. Permutational multivariate analysis of variance (PERMANOVA) with 999 permutations was carried out to determine significant differences in the physical parameters and chemical and nutritional parameters, significant differences in the community structures of Cave, Reservoir, Stream, and Null model communities. The analysis was performed using the “adonis” function of the “vegan” package and the “p.adjust” function of the “stats” package. Principal co‐ordinates analysis (PCoA) was used to visualize the differences using the “vegan” package. To analyze the relationships between biodiversity and network topology, a linear regression was performed using the “lm” function of the “stats” package to examine the relationships between taxon richness and both the diameters and APLs of network modules. Additionally, a nonlinear regression analysis employing the “loess” method was conducted to evaluate how taxon losing ratio influences four network structural properties: overall network diameter, overall network APL, average module diameter, and average module APL. The Kruskal‐Wallis test was employed to assess the differences in degree and betweenness across taxa with different dominance levels, and the differences among the following three pairwise dissimilarities: those between Cave and Null model communities, between Reservoir and Null model communities, and between Stream and Null model communities.

## 3 Results

### 3.1 Habitat Heterogeneity and Biodiversity in the CRS Ecotone

**FIGURE 2:**
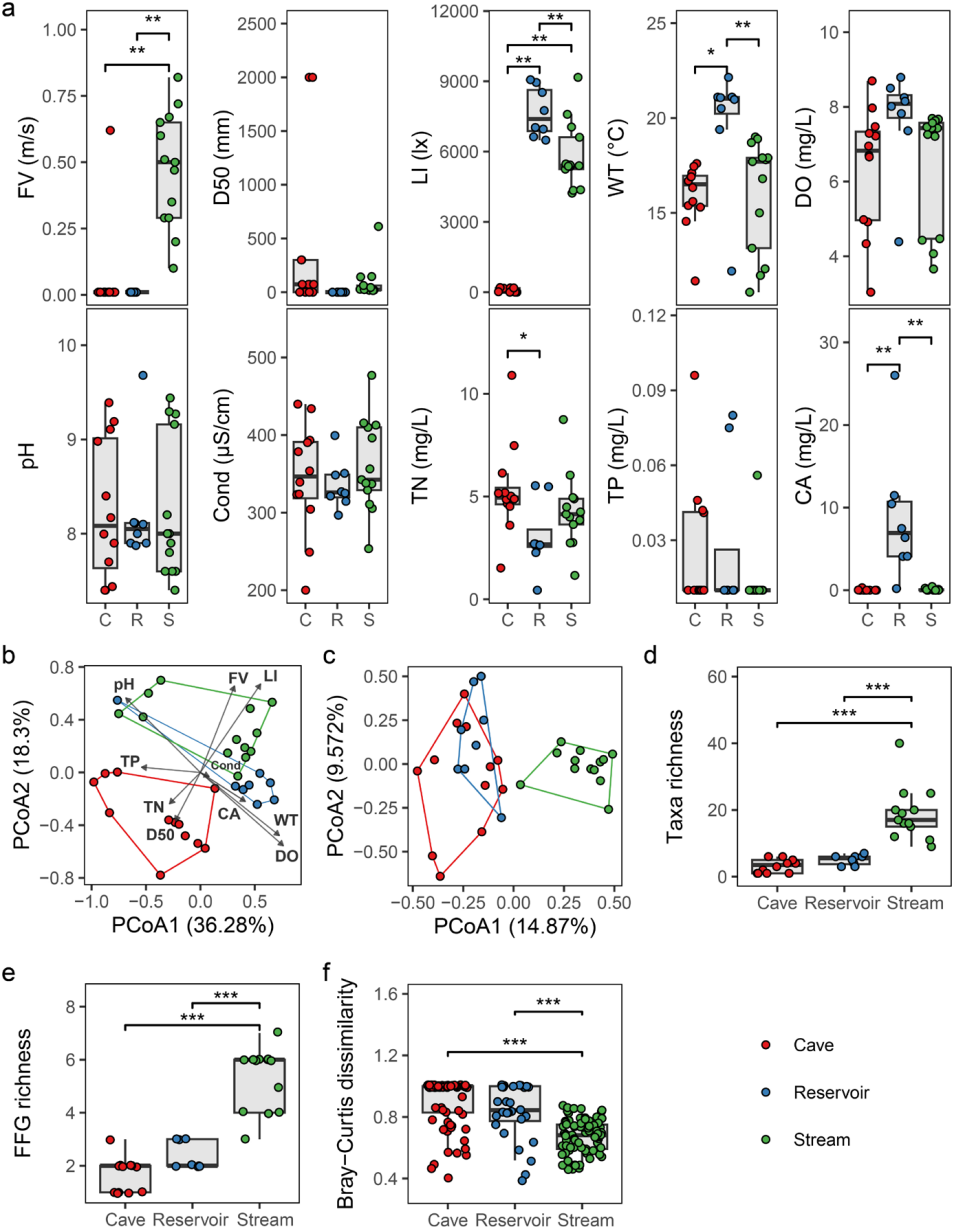
Habitat conditions and bio‐diversity indices for the CRS ecotone. (a) Value ranges of environmental variables of the sample sites (CA, chlorophyll a; Cond, conductivity; D50, median substrate grain size; DO, dissolved oxygen; FV, flow velocity; LI, light intensity; pH; TN, total nitrogen; TP, total phosphorus; WT, water temperature). (b) Habitat heterogeneity shown by: Principal coordinate analysis (PCoA) ordination of the physical, chemical and nutritional environmental variables (FV, D50, LI, WT, DO, pH, Cond, TN, TP and CA). (c) PCoA ordination of macroinvertebrate taxa. The CRS ecotone diversity shown by: α diversity including (d) taxa richness, (e) functional feeding group (FFG) richness, and (f) β diversity. Significant test: *p < 0.05, **p < 0.01, ***p < 0.001.

Figure 2a shows variations in flow velocity (FV), light intensity (LI), water temperature (WT), total nitrogen (TN) and chlorophyll‐a (CA) clear differences (ANOVA) in their values (the statistical results see Table S1 in the supplemental file). PCoA revealed significant distinction (*p* = 0.001, PERMANOVA) in the physical parameters—FV, D50 and LI—among Cave, Reservoir and Stream (Figure 2b). Compared with Cave or Reservoir, more discrete points of Stream showed that the dissimilarity among physical parameters of Stream sites was higher than that of Cave and Reservoir, reflecting higher habitat heterogeneity. However, there was no significant difference (*p* = 0.345, PERMANOVA) among chemical and nutritional parameters such as dissolved oxygen (DO), pH, conductivity, CA, TN and total phosphorous (TP). Overall, the Cave showed environmental conditions which characterized by relatively low flow velocity, considerable variability in the substrate particle size, low light intensity, high levels of total nitrogen and total phosphorus, yet low chlorophyll‐a concentration. Reservoir was typically marked by low flow velocity, uniform substrate particle size, and high concentrations of total nitrogen, total phosphorus and chlorophyll‐a. In contrast, Stream displayed high flow velocity and low concentrations of total phosphorus and chlorophyll a.

A total of 120 macroinvertebrate taxa were collected from the CRS ecotone. Among them, 23 taxa occurred in Cave, where Diptera was the most dominant order (13 taxa), followed by Tubificida (5 taxa) and Odonata (2 taxa). In Reservoir, 25 taxa were collected, with Diptera (16 taxa) and Tubificida (3 taxa) being the predominant orders. Total 100 taxa were identified in Stream; Diptera (45 taxa), Trichoptera (11 taxa) and Coleoptera (10 taxa) were the dominant orders. In terms of biodiversity of CRS ecotone, there were reverse trends between Stream and Cave or Reservoir. Indeed, both taxa richness and FFG richness were significantly higher (*p* < 0.001, ANOVA) for Stream than for Cave or Reservoir, while there was no significant difference between Reservoir and Cave (Figure 2d,e, Table S2). However, the β diversity (Bray–Curtis dissimilarity) among sites within Stream was significantly lower (*p* < 0.001, ANOVA) than that observed in either Cave or Reservoir (Figure 2f), and the taxa composition of Stream showed higher similarity than the Cave or Reservoir which also indicated by the PCoA space (Figure 2c). It was suggested that despite higher habitat heterogeneity of Stream sites, their exhibited significantly lower β diversity.

### 3.2 Co‐Occurrence Networks and Modules in Maintaining Biodiversity

**FIGURE 3:**
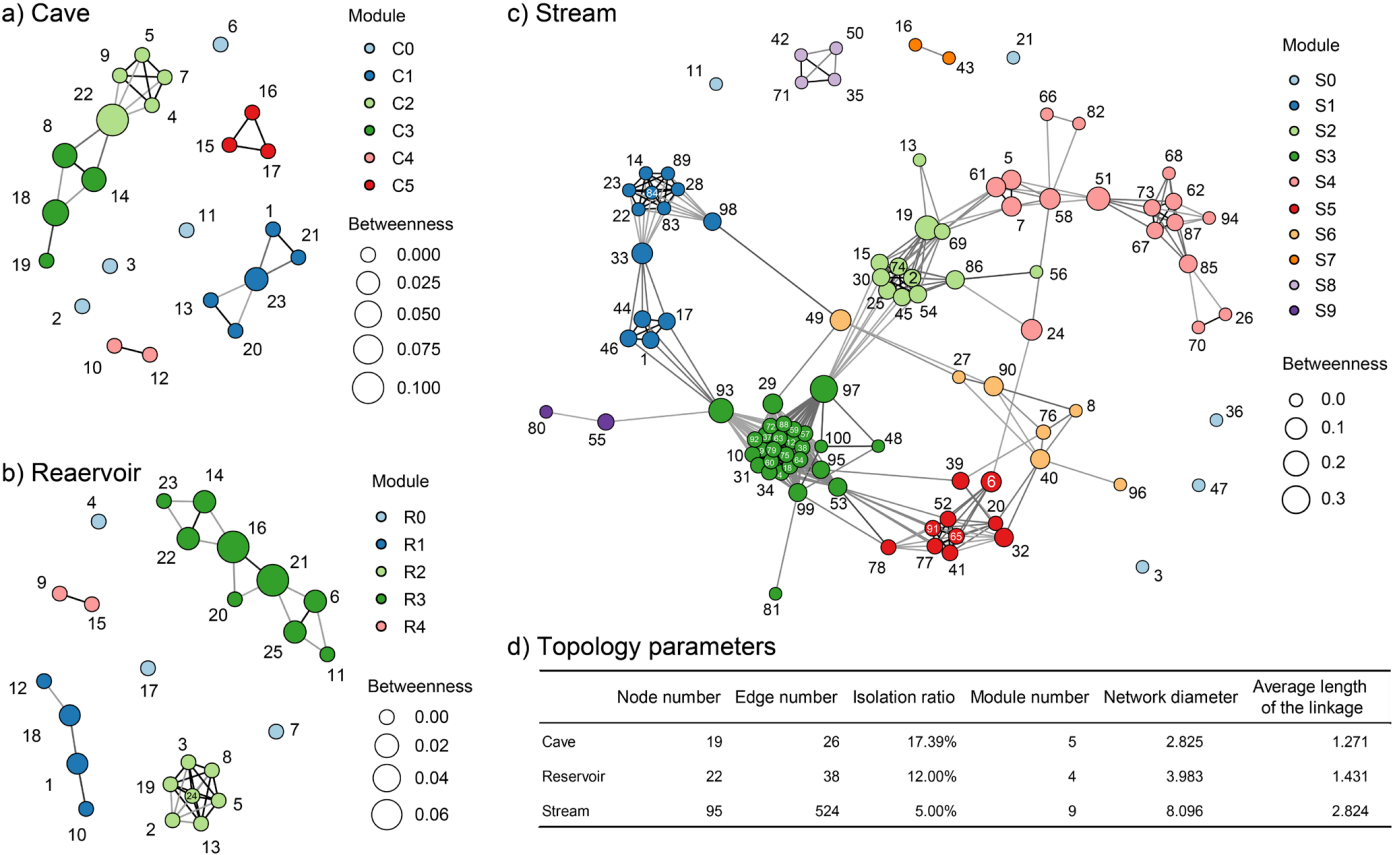
Macroinvertebrate co‐occurrence networks of (a) Cave, (b) Reservoir, and (c) Stream. Nodes with same color belonging to the same module. C0: Isolated taxa of Cave, C1—C5: Modules 1–5 of Cave; R0, R1—R4: Isolated taxa and modules 1–4 of Reservoir; S0, S1—S9: Isolated taxa and modules 1–9 of Stream. Linkages showing the potential connections between two nodes and sizes of nodes showing their betweennesses. Taxa of the nodes labeled by serial numbers were list in Table S3. (d) The network topology parameters for Cave, Reservoir and Stream.

Co‐occurrence networks (Figure 3) showed strong relationships among taxa across the communities in the CRS ecotone. Co‐occurrence network of Stream was larger (diameter = 8.10, APL = 2.82) than those of Cave (diameter = 2.82, APL = 1.27) and Reservoir (diameter = 3.98, APL = 1.43) (Figure 3d). The co‐occurrence network in Stream was more intact, while Cave and Reservoir exhibited higher isolation ratios (Figure 3d). Stream had about 5.0% isolated taxa, including *Antocha*, *Cricotopus* and *Lebertia*, Cave had about 17.4% isolated taxa, including *Branchiura*, *Caenis* and *Limnodrilus*, and Reservoir had about 12.0%, including *Caenis*, *Coelotanypus*, and *Micronecta* (Table S3). The Stream network was composed of nine modules, which were interconnected with each other, thus linking most of the nodes (Figure 3c). The Reservoir network comprised four modules, characterized by weak inter‐module connections and strong intra‐module node connections (Figure 3b). Lastly, the structure of the Cave network was relatively loose, comprising five modules, each containing few nodes (Figure 3a).

**FIGURE 4:**
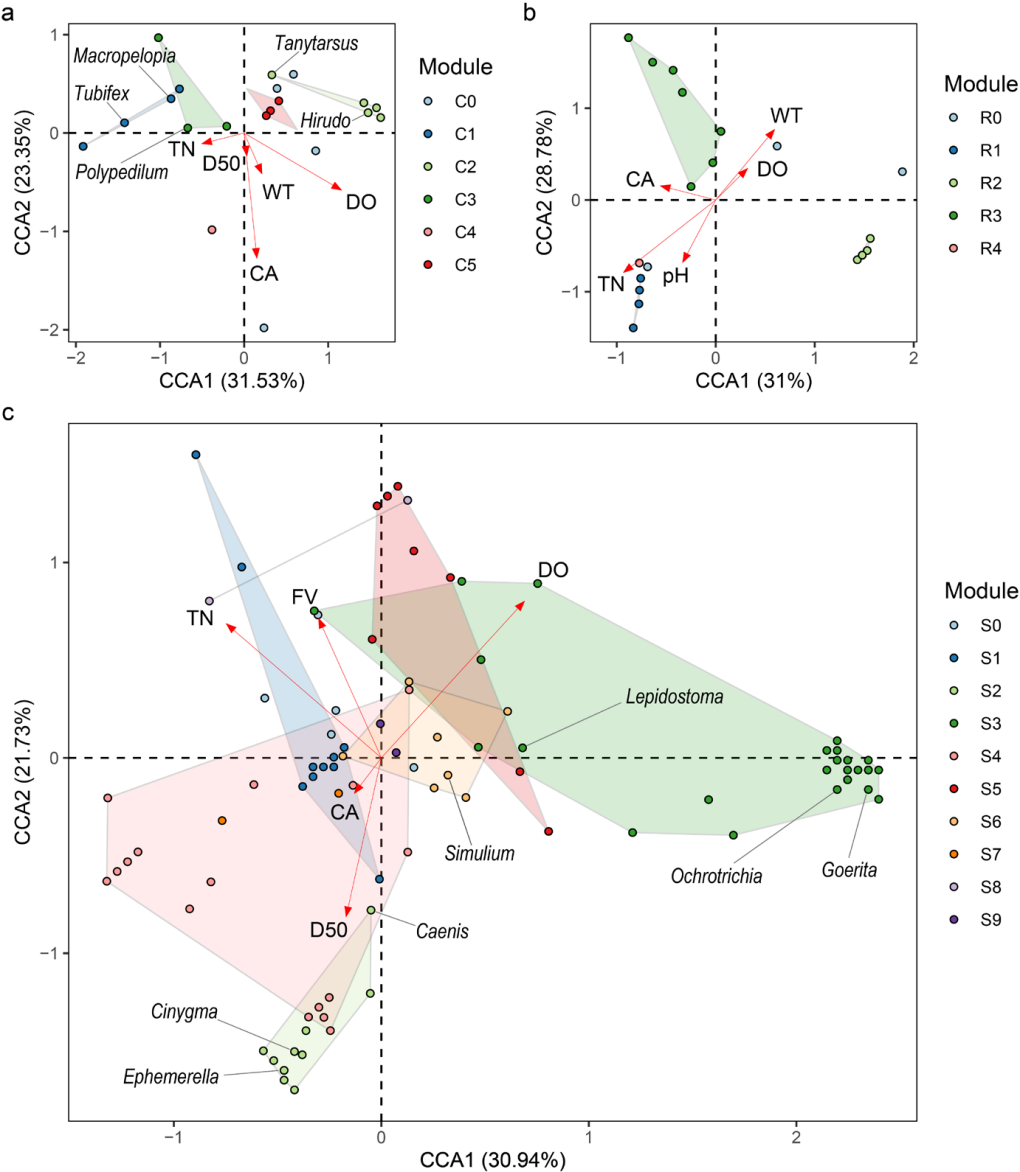
Canonical correspondence analysis (CCA) of modules in (a) Cave, (b) Reservoir and (c) Stream. Environmental parameters were abbreviated as the same in Figure 2 and modules were abbreviated as the same in Figure 3.

The Cave and Reservoir co‐occurrence networks as shown by distinct modules were clearly distinguished in CCA ordination spaces (Figure 4a,b). In Cave, DO and CA were the most important environmental factor in shape macroinvertebrate community structures. The modules C1 and C3 showed negative relations with DO and 66.7% taxa of them were Chironomidae and Naididae which could tolerate hypoxia, such as *Macropelopia*, *Polypedilum*, *Tubifex* etc. On the contrary, taxa in C2 including *Hirudo* and *Tanytarsus* preferred substrate with high DO. In Reservoir, WT and TN played relative more important roles. Above all, taxa within the same module might prefer similar habitat environments. In CCA space of Stream (Figure 4c), the modules S2, S6, S7, S8 and S9 demonstrate mutual overlap. Each of the dissolved oxygen, total nitrogen, flow velocity and substrate particulate size played pivotal roles in the formation and shaping of the community structure. The taxa in S2 showed a positive relationship with D50, such as *Caenis*, *Cinygma* and *Ephemerella* etc. The S3 showed a positive relationship with DO while a negative relationship with TN, where 35.7% taxa were EPT taxa adapted to clear water with high DO, such as *Nemoura*, *Ephemera* and *Rhyacophila*. Compared with S3, taxa in the S5, such as *Galba*, *Corbicula* and *Lumbriculus* preferred habitat with high DO and were tolerant to high TN.

**FIGURE 5:**
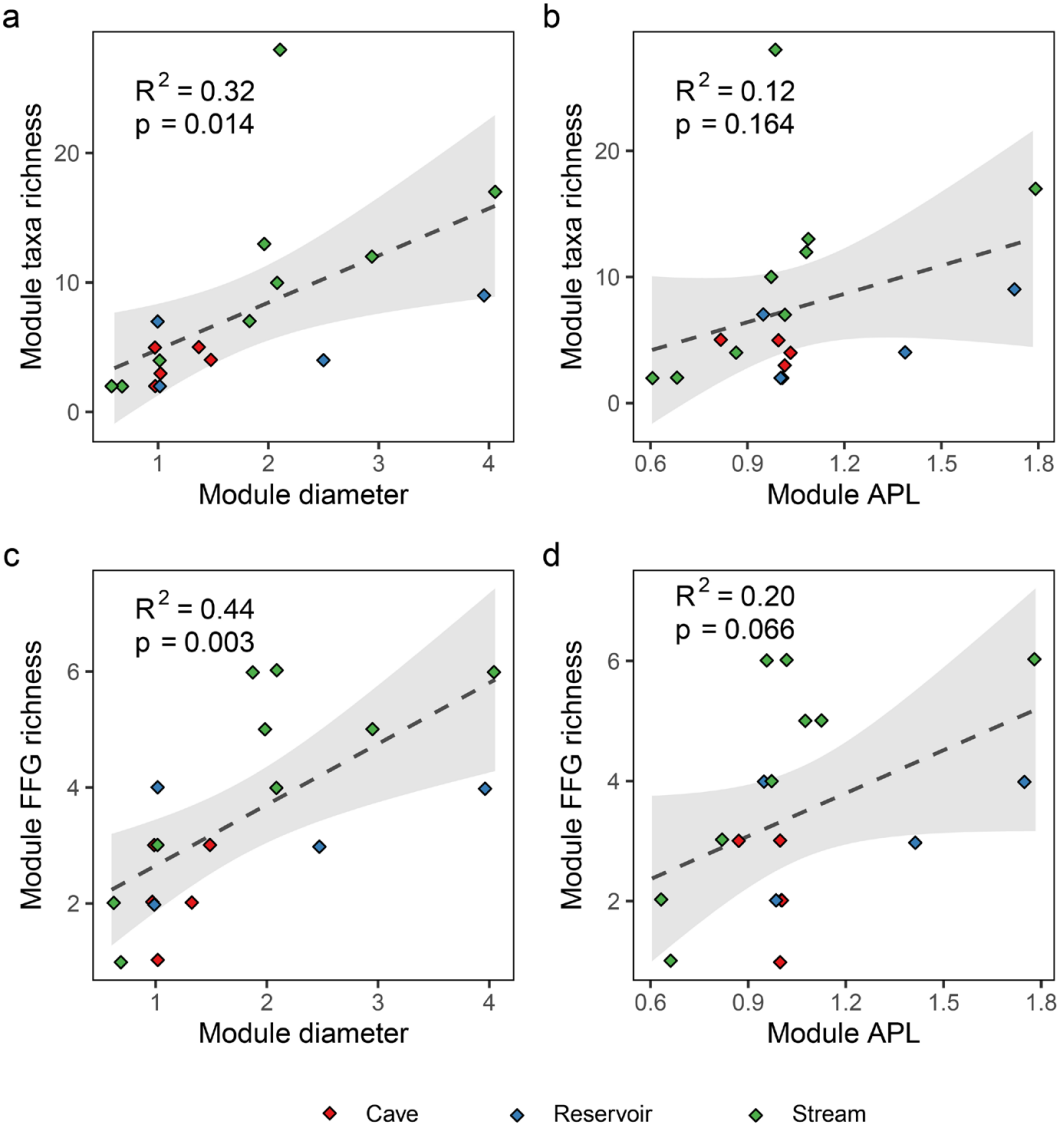
Relationships between module taxa richness and (a) module diameter and (b) module average path length (APL); and between Functional Feeding Group (FFG) richness and (c) module diameter and (d) module APL.

There was a significant positive correlation between taxa richness and diameter of modules (*R* ^2^ = 0.32, *p* = 0.014) (Figure 5a), and a potential positive relationship between taxa richness and APL of modules (*R* ^2^ = 0.12, *p* = 0.164) (Figure 5b). Consistent with this, a clear positive relationship was observed between diameter and FFG richness (*R* ^2^ = 0.44, *p* = 0.003) (Figure 5c), while a marginally significant relationship was identified between APL and FFG richness (*R* ^2^ = 0.20, *p* = 0.066) (Figure 5d).

### 3.3 Keystone Taxa in Shaping Biodiversity

**FIGURE 6:**
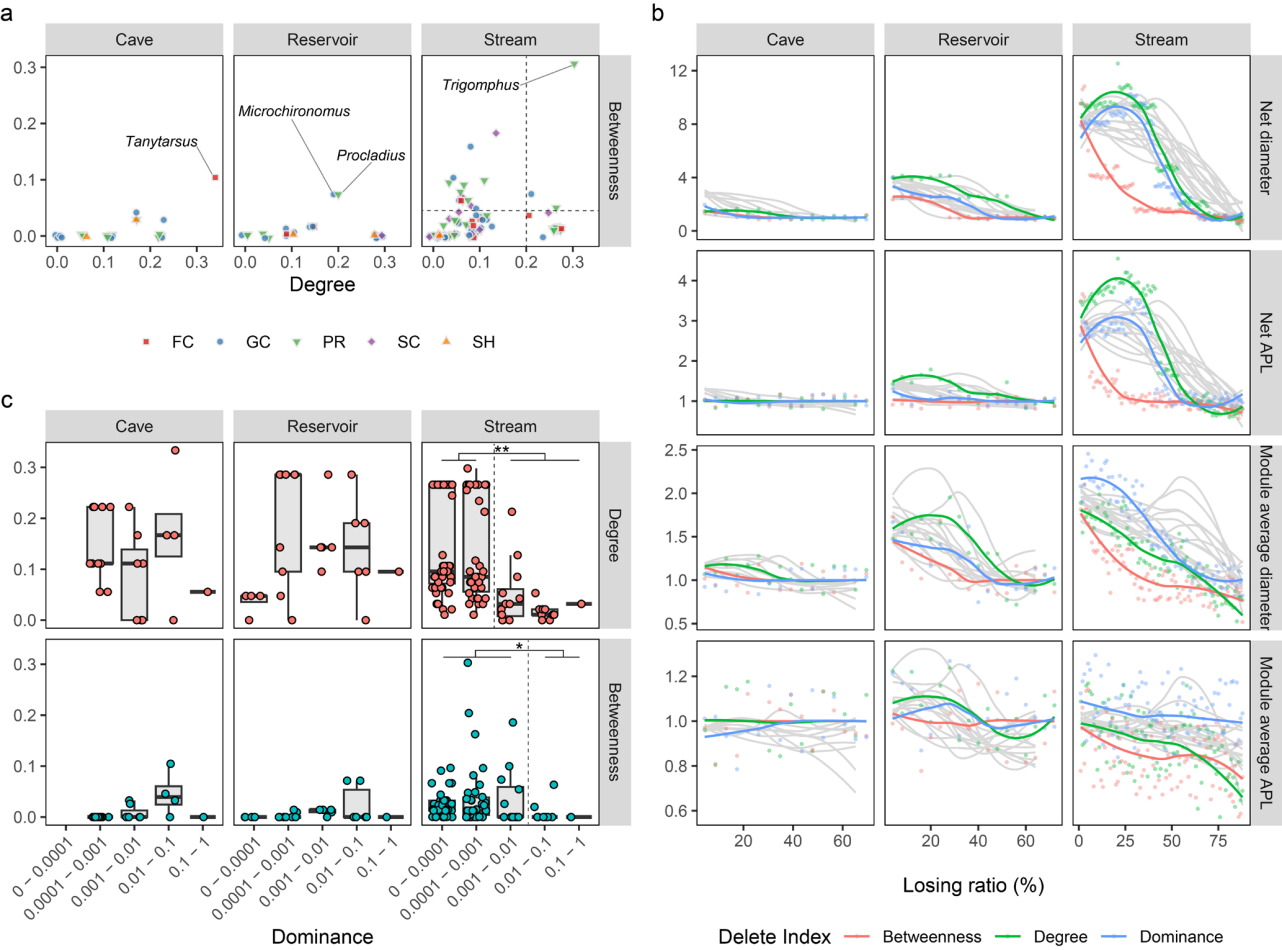
(a) Relationships between degree and betweenness of macroinvertebrates in the CRS ecotone. (b) Net diameter, net average link length (APL), module average diameter and module average APL decreasing with increasing taxa loss ratio. The red, green, blue and gray lines indicated trends for scenarios that taxa lost following descending order of their betweenness, degree, dominance and lost randomly. The fittings of lines are presented in Figure S1. (c) Degrees and betweenness of taxa with different dominance levels. Significant test: *p < 0.05, **p < 0.01.

Keystone taxa which had higher centrality (higher degree or higher betweenness) were critical for modules and co‐occurrence networks and played important roles in determining biodiversity. In Cave and Reservoir, most nodes were directly connected to other nodes or form small modules, so their betweenness (0.011 ± 0.025 for Cave, 0.009 ± 0.020 for Reservoir) were clear lower (*p* < 0.001) than Stream (0.029 ± 0.047), while their degrees were similar (0.126 ± 0.089, 0.145 ± 0.103, and 0.112 ± 0.094 for Cave, Reservoir, and Stream, respectively) (Figure 6a). In Cave, a FC taxon *Tanytarsus* had the highest degree (0.333) and the highest betweenness (0.105). PR had similar degrees (0.139 ± 0.066, *p* = 0.2) with GC (0.094 ± 0.091). In Reservoir, *Microchironomus* (GC) and *Procladius* (PR) were identified as keystone taxa, with higher degrees (0.190) and higher betweenness (0.071). However, the degrees of PR were lowest (0.071 ± 0.078) in FFGs. Compared with Cave and Reservoir, PRs were more important for Stream. They have the characteristics of higher degree (0.131 ± 0.102) and higher betweenness (0.037 ± 0.059) than other FFGs. Particularly, in taxa with degree higher than 0.2, there were 38.5% taxa were PR, that was much higher than other FFGs, such as 23.1% for GC, 19.2% for SC, 7.7% for FC, 7.7% for SH. Then in taxa with betweenness higher than 0.05, the ratio of PR, GC, SC and FC was 43.8%, 25.0%, 18.8% and 6.3% respectively. The most important keystone taxon was *Trigomphus* which was a PR with degree of 0.298 and betweenness of 0.303.

Loss of keystone taxa would cause remarkable decrease in diameters and APLs of co‐occurrence network and modules. In Stream, with taxa losing successively following descending order of their betweenness, diameter and APL of co‐occurrence network, average diameter and APL of modules all declined rapidly (Figure 6b). The loss of high betweenness taxa caused a more remarkable reduction than the loss of high degree taxa, high dominant taxa and randomly extinction did. When 25% highest betweenness taxa lost, diameter of co‐occurrence network cut almost a half, meanwhile only until more than half of the dominant taxa lost, significant decrease of diameters and APLs were identified. In addition, the loss of high degree taxa might cause more serious impact than that of high dominant taxa or randomly extinction. As larger diameter or APL of co‐occurrence network and modules may support higher taxa richness and FFG richness (Figure 2d,e, Figure 5), extinction of keystone taxa would cause more severe biodiversity degradation. At last, keystone taxa loss altered co‐occurrence network structures depending on environmental conditions. For more complex network in Stream than Cave and Reservoir, the losing of keystone taxa induced stronger collapse.

Nevertheless, the keystone macroinvertebrate taxa did not necessarily correspond to those with the highest dominance. In Stream, the dominant taxa (dominance > 0.001) had a significantly (*p* < 0.001, Kruskal‐Wallis test) lower degree than the other taxa (dominance < 0.001) (Figure 6c). The rare taxa (dominance < 0.01) also showed significantly (*p* = 0.02, Kruskal‐Wallis test) higher betweenness than the taxa with higher dominance (> 0.01). In Cave and Reservoir, the dominant taxa (dominance > 0.1) also showed relative lower degree and lower betweenness. *Trigomphus* in Stream (ranked 27th among 102 taxa), *Tanytarsus* in Cave (5th/23 taxa) and *Procladius* in Reservoir (7th/25 taxa) were the top keystone taxa.

### 3.4 Stochastic Processes in Shaping and Maintaining Macroinvertebrate Diversity

**FIGURE 7:**
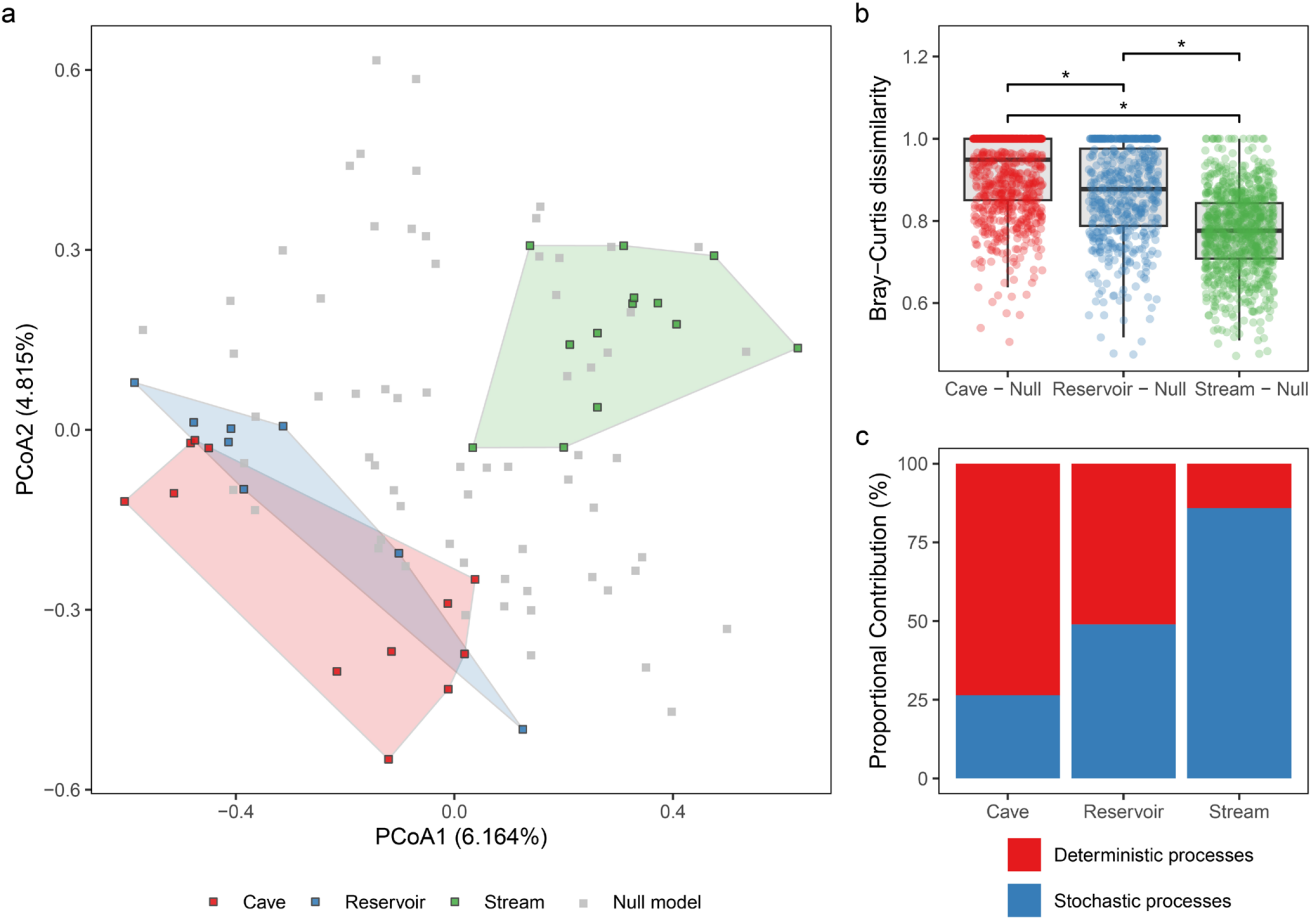
(a) Principal coordinate analysis (PCoA) of the macroinvertebrate communities in the CRS ecotone and null model communities. (b) A comparative analysis of Bray–Curtis dissimilarity among three aquatic ecosystems: Cave vs. Null model (Cave—Null), Reservoir vs. Null model (Reservoir—Null), and Stream vs. Null model (Stream—Null). Significant test: *p < 0.05. (c) Proportional contribution of deterministic processes and stochastic processes.

The comparison between communities of the CRS ecotone and communities simulated based on null model showed that Stream communities were more similar with randomly communities, yet Cave communities and Reservoir communities were more different with randomly distributed communities. PCoA (the first two axes accounting for 11.0% of the total variance) showed a significant difference (*p* < 0.05, Kruskal‐Wallis test) in macroinvertebrate community structure in Cave vs. Null model or Reservoir vs. Null model, and Stream did not show a significant difference with Null model (Figure 7a). The Bray–Curtis dissimilarity between Cave communities and Null model communities was significantly (*p* < 0.05, Kruskal‐Wallis test) greater than that between Reservoir communities and Null model communities, which in turn showed remarkably higher dissimilarity (*p* < 0.05) compared to Stream communities and Null model communities (Figure 7b).

The NST analysis (Figure 7c) shows that Cave was predominantly governed by deterministic processes, accounting for 73.6% of the observed proportional contribution. In contrast, Stream was primarily influenced by stochastic processes, which contributed 85.9%. The Reservoir exhibited a nearly balanced contribution from both processes, with deterministic and stochastic processes accounting for 51.1% and 48.9%, respectively.

## 4 Discussion

### 4.1 Role of Habitat Heterogeneity in Shaping Macroinvertebrate Diversity in the CRS Ecotone

The CRS ecotone in southwest China clearly exhibited distinct macroinvertebrate communities and ecosystem functioning, driven by fundamentally different environmental conditions. Cave, characterized by perpetual darkness and stable physicochemical conditions, hosted a lower α diversity and specialized community. Limited trophic linkages and increased hypoxic stress among Cave macroinvertebrate community in the region clearly indicate evolutionary filters they have that determine their functional diversity and ecosystem (Pacioglu et al. 2021). In the reservoir, a lentic and open system, displayed an intermediate α diversity and intermediate β diversity, where nutrient concentration and water temperature should have played the most critical roles in determining taxa tolerance (Santos et al. 2022). Unlike other two, the stream ecosystem was shaped by hydrological energy with highly variable flow supporting increased α diversity and low β diversity, where flow regime and substrate heterogeneity should have been the important drivers of the macroinvertebrate community structure (Duan et al. 2009; Zhou et al. 2023).

### 4.2 Roles of Keystone Taxa

This study reveals that keystone taxa with high centrality in communities are the fundamental units for the maintenance of biodiversity, which have connected directly or indirectly to many other taxa. The extinction of keystone taxa (especially a small number of taxa with highest betweenness such as *Trigomphus*) can rapidly reduce network sizes, include network diameter, network APL, module average diameter and module average APL, which are associated with taxonomic diversity and functional diversity (Figure 5). Vanishing of keystone taxa may cause modules and networks to be disassembled, resulting in a marked shift in the composition, structure, and functioning of communities (McClure 2019).

The keystone taxa in Stream predominantly occupy higher trophic positions, functioning as higher‐order predators that regulate macroinvertebrate community structure. There was a noticeable change in FFG of keystone taxa in the CRS ecotone (Figure 6a). From Cave to Reservoir to Stream, increasing number of keystone taxa were recorded as predators. In Stream, powerful higher‐order predator such as *Trigomphus*, *Macromia*, *Neochauliodes* and *Laccornellus* prey on insects, crustaceans, and even small fish (Pritchard 1964; Hayashi 1988; Culler et al. 2023). Higher‐order predators have ability to integrate the available energy resources in a variety of channels within the ecosystem, which are derived from the multiple consumer‐food chain sources at already existing habitats indifferent to each other (Rooney et al. 2006), that may be one of the reasons why higher‐order predators have higher betweenness. Some other studies have shown that top predators such as carnivorous stinkbugs can exert important effects over the diversity, structure and function of ecosystems (Gregr et al. 2020; Zhou et al. 2023), which suggests that such keystone taxa may play critical roles in multiple ecological processes.

Recently, increasing studies have shown that keystone taxa maximally achieve their ecological function irrespective of abundance and frequency (La Bella et al. 2024). In our study, the relationship between degree or betweenness centrality and dominance implies that numerically dominant taxa have lower centrality (Figure 6c), thereby supports the notion that keystone taxa in communities may be not dominant taxa. The limited connectivity of dominant taxa suggests that they have less interaction with other taxa and they may receive limited influence from other taxa. Usually, macroinvertebrate communities with higher ratios of dominant taxa show lower diversity (Liu et al. 2019). In addition, according to energy flow patterns in food webs, higher‐order predators usually have lower biomass than their prey (Zhou et al. 2024).

### 4.3 Deterministic Processes

Co‐occurrence network modules emerge through topological interactions between keystone taxa and peripheral taxa, represent important co‐evolutionary ecological units of macroinvertebrate communities, which are the basic organizational form for maintaining diversity (Simons et al. 2019). In response to habitat disintegration, pairwise interactions can lead to competitive exclusion of species. However, higher‐order species interactions play a pivotal role in sustaining relatively high diversity by weakening the destabilizing effect of pairwise interactions (Bairey et al. 2016). In our study, Stream shows larger APLs and diameters in modules and co‐occurrence networks than Cave and Reservoir which mean taxon‐taxon interactions need more intermediate taxa (Figure 3). These observed positive relationships between larger diameters or longer APL and higher α diversity suggests enhanced multi‐species interactions which are conducive to maintaining species coexistence.

On the other hand, macroinvertebrates respond to abiotic environments as modules, and this form of community structure may potentially facilitate taxa coexistence, thereby enhancing biodiversity (Bray et al. 2018). In Stream, interactions within modules may have extended the survival of macroinvertebrates, and some taxa might further alter their habitat preferences. For instance, *Caenis*, *Cinygma* and *Ephemerella* in the S2 are typical EPT taxa that prefer high DO and high FV, yet the S2 showed negative relationships with DO and FV (Figure 4c). Other genera, *Simulium* in the S6 prefer typically to inhabiting the lotic environments (Schmidt‐Kloiber and Hering 2015), however the S6 showed a negative relationship with FV. Trichoptera generally prefer a higher substrate particle size (Allan and Castillo 2007), however S3 site showed a negative correlation with D50, which included several types of Trichoptera, such as *Goerita*, *Lepidostoma*, *Ochrotrichia* etc. Therefore, biological interactions among modules may enhance taxa tolerance to environmental stress (Germain et al. 2018). Bray et al. (2018) also identified that beside environmental stress itself, taxa can be influenced by interactions with organisms which are tolerant or sensitive to environmental stress.

Modules are connected to each other to form a network, which further affects diversity. In reality, the co‐occurrence networks of Cave and Reservoir would become sub‐networks of Stream due to the resulted of the absence of certain intermediate nodes such as *Trigomphus*, *Macromia* and *Neochauliodes* (nodes 97, 53, and 58 in Figure 3c, respectively). Compared with Stream, fragmentation of networks of Cave and Reservoir were recorded high, as a result both module size and network size declined, leading to weaken higher‐order interactions and decreased betweenness of taxa. The condition may further explain a decline in biodiversity of Cave and Reservoir in the karst environment of southwest China.

### 4.4 Stochastic Processes

Beside deterministic processes of biotic and abiotic environmental factors, this study demonstrates that stochastic processes play a stronger role in determining diversity and structuring Stream macroinvertebrate community, and supporting the universal hypothesis ‘local environmental conditions determine roles of stochastic and deterministic processes’ (Dini‐Andreote et al. 2015). Our results showed increased likelihoods of stochastic processes in the karst environment of southwest China significantly influencing Stream macroinvertebrate diversity and communities (85.9%). In the case of deterministic processes, which were associated with environmental factors predominantly observed in Cave and Reservoir had 73.6% and 51.1%, contributions (Figure 7c). In Cave and Reservoir CCA plots (Figure 4), modules showed clear relationships with environmental factors, and the environmental gradient demonstrated effective sorting of taxa. In contrast, the overlapping observed among modules in the Stream plot showed relatively weaker deterministic processes of environmental factors on community structure, indicating that the stream macroinvertebrate community was not solely shaped by environmental conditions alone. These findings suggest that Stream showed the strongest stochastic influences, followed by Reservoir, while Cave demonstrated the lowest reliance on stochastic assembly mechanism (Figure 7).

Stochastic processes can shape diversities by dynamic life history characteristics such as dispersal limitation, ecological drift, colonization and extinction (Chase 2010). Dispersal processes of macroinvertebrates include active dispersal (self‐movement) and passive dispersal, relying mostly on external environmental forces such as water and wind (Li et al. 2019). Compared to Stream, slower flow velocities in Cave (mean FV = 0.06 m/s) and Reservoir (0.01 m/s) likely to have reduced ecological stochasticity in these habitats (Li et al. 2020). Algae are considered as higher quality food source for macroinvertebrates than terrestrial organic matter (Guo et al. 2016). However, due to the absence of light (LI = 66.3 lx), Cave primarily contained organic matter and very little algal content (CA = 0.028 mg/L) (Table S1). This constrained availability of trophic resources likely resulted in lower biodiversity in Cave, despite clear differences in substrate composition across different Cave sites (D50 = 407.7 mm). The more suitable environmental conditions for macroinvertebrates, such as the availability of a characteristically rich habitat type and a high biomass production, the stochastic processes would become important. For instance, under extreme environmental conditions, e.g., at high stream flow, factors causing severe stress would become critical in influencing community structures and distribution of macroinvertebrates (Zhou et al. 2017). Li et al. (2019) demonstrated that deterministic processes (such as environmental filtering) would become active in harsh environmental conditions, like glacier‐fed streams, but stochastic processes would reduce in less harsh environment.

### 4.5 Implications for Management and Conservation Practices

Both Reservoir and Cave in the CRS ecotone are important ecosystems in karst landscapes in southwest China, although they showed relatively weak performance in α biodiversity and co‐occurrence network structure. The Cave possess very distinct environmental characteristics, constituting critically significant habitats for the survival of certain types of endangered macroinvertebrate species. McNie and Death (2017) argued that natural subterranean systems are usually more resistant to various types of stressors including drought and floods from the earth's surface and less affected by catchment changes caused by both climate change and human disturbances. Reservoir on the other hand, provides suitable habitats for algae (Xie et al. 2025) with rich diversity and abundance thereby strengthening the base of the food web (Guo et al. 2016). Algae also enhance important trophic resources for macroinvertebrates in Cave and Stream and further increases ecological stability in ecotone system. The presence of Cave and Reservoirs in the CRS ecotone is beneficial for the intact biological diversity and protection of endangered flora and fauna due to increased habitat heterogeneity (Thomsen et al. 2022). Suitable environmental conditions in the Stream habitat favor rich biodiversity and ecosystem services such as regulating services (e.g., nutrient cycling and water purification) (Kattel 2022). Keeping flow system connected among Cave, Reservoir, and Streams would maintain ecosystems and biodiversity intact within the CRS ecotone and generate range of different ecosystem services (Nayeli Luis‐Vargas et al. 2024). Above all, comprehending the drivers that influence biodiversity in the CRS system, as well as their effects on biodiversity patterns, are useful for determining resource allocations and efforts for protecting biodiversity in the ecotone region.

## 5 Conclusion

This study assessed environmental characteristics and the status of biodiversity in the CRS ecotone and identified the key drivers regulating the regional biodiversity. In the CRS ecotone, Stream demonstrated the highest taxonomic diversity and functional diversity yet the lowest β diversity, whereas Cave exhibited the lowest α diversity but the highest β diversity. We found that keystone taxa and co‐occurrence network modules were the fundamentally significant for maintaining macroinvertebrate biodiversity in CRS ecotone in southwest China. Keystone taxa, such as *Tanytarsus* in Cave, *Procladius* in Reservoir, *Trigomphus* in Stream, underpinned diversity and community assembly by linking with other taxa to form modules and networks. Modules enhanced interactions among macroinvertebrates and promoted species coexistence which further influences diversity. Under suitable environmental conditions, as exemplified by Stream, stochastic processes played a stronger role contributing to as high as 85.9% in the maintenance of diversity than deterministic processes. Conversely, in resource‐limited environments such as Cave, deterministic processes would become the main driving force with 73.6% contribution, leading to keystone taxa loss and modules disconnections. However, the lack of temporal replications, such as the absence of seasonal samplings, prevented a comprehensive understanding of the significant temporal effects on species dynamics in CRS ecotone of the karst landscape in southwest China. Secondly, this study focused only on functional feeding traits, neglecting other functional traits, which may have restricted broader applicability of the findings. Future studies should address these limitations by conducting multi‐seasonal assessments and integrating broader ecological interactions to enhance the validity and applicability of the results.

## Author Contributions

**Wei Liu:** conceptualization (equal), data curation (lead), formal analysis (lead), investigation (lead), methodology (lead), software (lead), visualization (lead), writing – original draft (lead), writing – review and editing (supporting). **Mengzhen Xu:** conceptualization (lead), formal analysis (supporting), funding acquisition (lead), methodology (supporting), project administration (lead), resources (lead), software (supporting), supervision (lead), writing – original draft (equal), writing – review and editing (lead). **Giri R. Kattel:** writing – original draft (equal), writing – review and editing (equal). **Xudong Fu:** writing – original draft (equal), writing – review and editing (equal).

## Funding

This work was supported by State Key Laboratory of Hydroscience and Engineering (sklhse‐TD‐2024‐E01). National Natural Science Foundation of China (U2243222).

## Conflicts of Interest

The authors declare no conflicts of interest.

## Supporting information


**Data S1:** ece372802‐sup‐0001‐supinfo.docx.

## Data Availability

The data supporting the findings of this study and the R code scripts for generating the figures are available in figshare at: https://figshare.com/s/4ce8ee27b5e83aeb2b36
