# Supplementary material for: Deterministic and Stochastic Processes Regulate Co‐Occurrence Network Structure and Shape Macroinvertebrate Diversity in Karst Environment
Source: Ecol Evol. 2026 Jan 6;16(1):e72802. doi: 10.1002/ece3.72802 (PMC12774801; doi:10.1002/ece3.72802)
Supplement: Supplementary file 1 — Data S1: ece372802‐sup‐0001‐supinfo.docx. [file ECE3-16-e72802-s001.docx]

**Deterministic and stochastic processes regulate co-occurrence network**

**and shape** **macroinvertebrate diversity in karst environment**

**Wei Liu**

*State Key Laboratory of Hydroscience and Engineering, Tsinghua University, Beijing 100084, China*

E-mail: iaoisora@hotmail.com

ORCID: https://orcid.org/0000-0001-8433-2640

**Mengzhen Xu ***

*State Key Laboratory of Hydroscience and Engineering, Tsinghua University, Beijing* *100084, China*

***** Corresponding author e-mail: mzxu@tsinghua.edu.cn

Tel: 0086-10-62788524

Fax: 0086-10-62772463

ORCID: https://orcid.org/0000-0002-2507-1935

**Giri R. Kattel**

*State Key Laboratory of Hydroscience and Engineering, Tsinghua University, Beijing 100084, China*

*Department of Infrastructure Engineering, The University of Melbourne,* *Parkville Victoria 3010, Australia*

E-mail: [giri.kattel@unimelb.edu.au](mailto:giri.kattel@unimelb.edu.au)

**Xudong Fu**

*State Key Laboratory of Hydroscience and Engineering, Tsinghua University, Beijing 100084, China*

E-mail: [xdfu@tsinghua.edu.cn](mailto:xdfu@tsinghua.edu.cn)


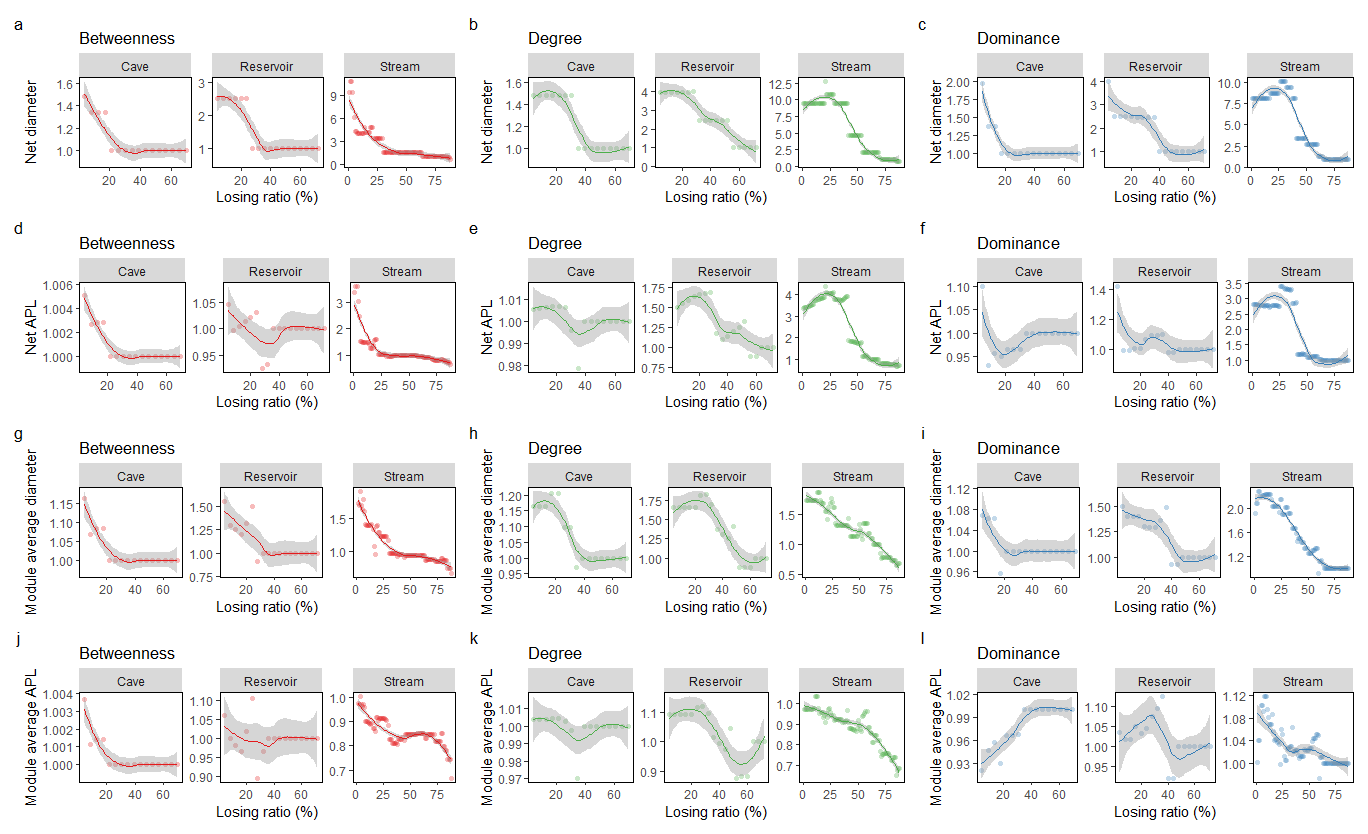


**Figure S1** The influence of taxa losing to (a), (b), (c) diameter of co-occurrence networks, (d), (e), (f) average link length (APL) of co-occurrence networks, (g), (h), (i) diameter of modules, (j), (k), (l) average link length of modules in the Cave-Reservoir-Stream (CRS) ecotone. In (a), (d), (g) and (j), genera lose descending order by their betweenness (Table S3); in (b), (e), (h) and (k), genera lose descending order by their degree; and in (c), (f), (i) and (l), genera lose descending order by their dominance. Shaded areas around dashed lines in (a) - (l) show 95% confidence intervals.

**Table S1** Environmental variables of the Cave-Reservoir-and-Stream (CRS) ecotone.

| **Environmental parameter** | **Abbreviation** | **Cave** | **Reservoir** | **Stream** |
| --- | --- | --- | --- | --- |
| Flow velocity  (mean ± SD, m/s) | FV | 0.061 ± 0.176 ^a^ | 0.01 ± 0.00 ^a^ | 0.475 ± 0.217 ^b^ |
| 50th percentile particulate size  (mean ± SD, mm) | D50 | 407.68 ± 751.17 ^a^ | 0.05 ± 0.00 ^a^ | 90.10 ± 163.09 ^a^ |
| Light intensity  (mean ± SD, lx) | LI | 66.3 ± 85.3 ^a^ | 7675.6 ± 1049.9 ^b^ | 5820.0 ± 1428.2 ^c^ |
| Water temperature  (mean ± SD, °C) | WT | 15.93 ± 1.71 ^a^ | 19.78 ± 3.26 ^b^ | 15.95 ± 3.01 ^a^ |
| Dissolved oxygen  (mean ± SD, mg/L) | DO | 6.35 ± 1.67 ^a^ | 7.7 ± 1.39 ^a^ | 6.46 ± 1.61 ^a^ |
| pH  (mean ± SD) | pH | 8.26 ± 0.74 ^a^ | 8.21 ± 0.60 ^a^ | 8.25 ± 0.75 ^a^ |
| Conductivity  (mean ± SD, µS/cm) | Cond | 344.2 ± 70.9 ^a^ | 335.4 ± 31.2 ^a^ | 360.3 ± 59.5 ^a^ |
| Total nitrogen  (mean ± SD, mg/L) | TN | 5.328 ± 2.246 ^a^ | 3.050 ± 1.692 ^b^ | 4.282 ± 1.804 ^b^ |
| Total phosphorus  (mean ± SD, mg/L) | TP | 0.025 ± 0.027 ^a^ | 0.027 ± 0.031 ^a^ | 0.014 ± 0.013 ^a^ |
| Concentration of chlorophyll a  (mean ± SD, mg/L) | CA | 0.028 ± 0.085 ^a^ | 8.772 ± 7.856 ^b^ | 0.073 ± 0.131 ^a^ |

Note: ^a^ and ^b^ indicate significant difference (p < 0.05) between groups. SD - standard deviation.

**Table S2** Biodiversity indices of the Cave-Reservoir-and-Stream (CRS) ecotone.

| **Diversity** | **Cave** | **Reservoir** | **Stream** |
| --- | --- | --- | --- |
| taxa richness  (mean ± SD) | 3.25 ± 2.01 ^a^ | 5 ± 1.51 ^a^ | 18.85 ± 7.99 ^b^ |
| FFG richness  (mean ± SD) | 1.67 ± 0.65 | 2.38 ± 0.52 | 5.31 ± 1.18 |
| β diversity  (mean ± SD) | 0.897 ± 0.165 ^a^ | 0.833 ± 0.179 ^a^ | 0.677 ± 0.108 ^b^ |

Note: ^a^ and ^b^ indicate significant difference (p < 0.05) between groups. SD - standard deviation.

**Table S3** Node traits of the co-occurrence network of three ecotone environments: (a) Cave, (b) Reservoir and (c) Stream. FFG is the functional feeding group trait of each taxon, including filter-collector (FC), gatherer-collector (GC), omnivore (OM), parasite (PA), piercer (PI), predator (PR), scraper (SC) and shredder (SH). The C0 are isolated taxa of Cave co-occurrence network and the C1-C5 are Cave modules 1-5, the R0 are isolated taxa and the R1-R4 are modules 1-4 in Reservoir, and the S0 are isolated taxa and S1-S9 are modules 1-9 in Stream.

1. Node traits of the Cave co-occurrence network

| **Ecotone** | **Taxon ID** | **Taxon** | **FFG** | **Module** | **Abundance**  **(ind.)** | **Dominance** | **Degree** | **Betweenness** |
| --- | --- | --- | --- | --- | --- | --- | --- | --- |
| Cave | 1 | *Bethbilbeckia* | PR | C1 | 4 | 0.00178 | 0.11111 | 0.00000 |
| Cave | 2 | *Branchiura* | GC | C0 | 5 | 0.00446 | 0.00000 | 0.00000 |
| Cave | 3 | *Caenis* | GC | C0 | 6 | 0.00802 | 0.00000 | 0.00000 |
| Cave | 4 | Ceratopogonidae | PR | C2 | 1 | 0.00045 | 0.22222 | 0.00000 |
| Cave | 5 | Chaoboridae | PR | C2 | 1 | 0.00045 | 0.22222 | 0.00000 |
| Cave | 6 | *Chironomus* | GC | C0 | 22 | 0.03922 | 0.00000 | 0.00000 |
| Cave | 7 | Culicidae | GC | C2 | 1 | 0.00045 | 0.22222 | 0.00000 |
| Cave | 8 | *Davidius* | PR | C3 | 4 | 0.00178 | 0.16667 | 0.03268 |
| Cave | 9 | *Hirudo* | PR | C2 | 1 | 0.00045 | 0.22222 | 0.00000 |
| Cave | 10 | *Lebertia* | PR | C4 | 2 | 0.00089 | 0.05556 | 0.00000 |
| Cave | 11 | *Limnodrilus* | GC | C0 | 7 | 0.00936 | 0.00000 | 0.00000 |
| Cave | 12 | *Macromidia* | PR | C4 | 1 | 0.00045 | 0.05556 | 0.00000 |
| Cave | 13 | *Macropelopia* | PR | C1 | 2 | 0.00089 | 0.11111 | 0.00000 |
| Cave | 14 | *Molophilus* | SH | C3 | 40 | 0.01783 | 0.16667 | 0.03268 |
| Cave | 15 | *Monopylephorus* | GC | C5 | 1 | 0.00045 | 0.11111 | 0.00000 |
| Cave | 16 | *Natarsia* | PR | C5 | 1 | 0.00045 | 0.11111 | 0.00000 |
| Cave | 17 | *Orthocladius* | GC | C5 | 1 | 0.00045 | 0.11111 | 0.00000 |
| Cave | 18 | *Paracladopelma* | GC | C3 | 13 | 0.01738 | 0.16667 | 0.04575 |
| Cave | 19 | *Polypedilum* | SH | C3 | 49 | 0.10918 | 0.05556 | 0.00000 |
| Cave | 20 | *Spirosperma* | GC | C1 | 2 | 0.00089 | 0.11111 | 0.00000 |
| Cave | 21 | *Tabanus* | PR | C1 | 4 | 0.00178 | 0.11111 | 0.00000 |
| Cave | 22 | *Tanytarsus* | FC | C2 | 13 | 0.01159 | 0.33333 | 0.10458 |
| Cave | 23 | *Tubifex* | GC | C1 | 6 | 0.00535 | 0.22222 | 0.02614 |

1. Node traits of the Reservoir co-occurrence network

| **Ecotone** | **Taxon ID** | **Taxon** | **FFG** | **Module** | **Abundance**  **(ind.)** | **Dominance** | **Degree** | **Betweenness** |
| --- | --- | --- | --- | --- | --- | --- | --- | --- |
| Reservoir | 1 | *Ablabesmyia* | GC | R1 | 35 | 0.00222 | 0.09524 | 0.00952 |
| Reservoir | 2 | *Branchiura* | GC | R2 | 320 | 0.01351 | 0.28571 | 0.00000 |
| Reservoir | 3 | *Bryophaenocladius* | GC | R2 | 32 | 0.00068 | 0.28571 | 0.00000 |
| Reservoir | 4 | *Caenis* | GC | R0 | 4 | 0.00008 | 0.00000 | 0.00000 |
| Reservoir | 5 | *Camptocladius* | GC | R2 | 16 | 0.00034 | 0.28571 | 0.00000 |
| Reservoir | 6 | *Chironomus* | GC | R3 | 272 | 0.00574 | 0.14286 | 0.01429 |
| Reservoir | 7 | *Coelotanypus* | PR | R0 | 8 | 0.00017 | 0.00000 | 0.00000 |
| Reservoir | 8 | *Dasyhelea* | GC | R2 | 48 | 0.00101 | 0.28571 | 0.00000 |
| Reservoir | 9 | *Elophila* |  | R4 | 1 | 0.00002 | 0.04762 | 0.00000 |
| Reservoir | 10 | *Lebertia* | PR | R1 | 10 | 0.00063 | 0.04762 | 0.00000 |
| Reservoir | 11 | *Limnodrilus* | GC | R3 | 408 | 0.01722 | 0.09524 | 0.00000 |
| Reservoir | 12 | *Limnophora* | PR | R1 | 1 | 0.00002 | 0.04762 | 0.00000 |
| Reservoir | 13 | *Limnophyes* | GC | R2 | 16 | 0.00034 | 0.28571 | 0.00000 |
| Reservoir | 14 | *Macropelopia* | PR | R3 | 120 | 0.00253 | 0.14286 | 0.01429 |
| Reservoir | 15 | *Mesosmittia* | GC | R4 | 3 | 0.00006 | 0.04762 | 0.00000 |
| Reservoir | 16 | *Microchironomus* | GC | R3 | 992 | 0.04188 | 0.19048 | 0.07143 |
| Reservoir | 17 | *Micronecta* | PR | R0 | 1136 | 0.02398 | 0.00000 | 0.00000 |
| Reservoir | 18 | *Mystacides* | GC | R1 | 7 | 0.00030 | 0.09524 | 0.00952 |
| Reservoir | 19 | *Physa* | SC | R2 | 16 | 0.00034 | 0.28571 | 0.00000 |
| Reservoir | 20 | *Polypedilum* | SH | R3 | 1594 | 0.20187 | 0.09524 | 0.00000 |
| Reservoir | 21 | *Procladius* | PR | R3 | 312 | 0.01317 | 0.19048 | 0.07143 |
| Reservoir | 22 | *Simulium* | FC | R3 | 8 | 0.00017 | 0.14286 | 0.01429 |
| Reservoir | 23 | *Tanytarsus* | FC | R3 | 483 | 0.02039 | 0.09524 | 0.00000 |
| Reservoir | 24 | *Tipula* | SH | R2 | 32 | 0.00068 | 0.28571 | 0.00000 |
| Reservoir | 25 | *Tubifex* | GC | R3 | 48 | 0.00101 | 0.14286 | 0.01429 |

1. Node traits of the Stream co-occurrence network

| **Ecotone** | **Taxon ID** | **Taxon** | **FFG** | **Module** | **Abundance**  **(ind.)** | **Dominance** | **Degree** | **Betweenness** |
| --- | --- | --- | --- | --- | --- | --- | --- | --- |
| Stream | 1 | *Ablabesmyia* | GC | S1 | 4 | 0.00008 | 0.05319 | 0.02602 |
| Stream | 2 | *Anodonta* | FC | S2 | 1 | 0.00002 | 0.10638 | 0.03258 |
| Stream | 3 | *Antocha* | GC | S0 | 136 | 0.01975 | 0.00000 | 0.00000 |
| Stream | 4 | Aspidytidae | PR | S3 | 1 | 0.00002 | 0.26596 | 0.01267 |
| Stream | 5 | *Assiminea* | SC | S4 | 1 | 0.00002 | 0.06383 | 0.06656 |
| Stream | 6 | *Atherix* | PR | S5 | 3 | 0.00012 | 0.06383 | 0.08191 |
| Stream | 7 | *Atrichops* | PR | S4 | 1 | 0.00002 | 0.06383 | 0.06656 |
| Stream | 8 | *Baetis* | GC | S6 | 770 | 0.19174 | 0.03191 | 0.00000 |
| Stream | 9 | *Bethbilbeckia* | PR | S3 | 2 | 0.00004 | 0.26596 | 0.01267 |
| Stream | 10 | *Bezzia* | GC | S3 | 1 | 0.00002 | 0.26596 | 0.01267 |
| Stream | 11 | *Branchiura* | GC | S0 | 17 | 0.00176 | 0.00000 | 0.00000 |
| Stream | 12 | *Brychius* | SC | S3 | 3 | 0.00006 | 0.26596 | 0.01267 |
| Stream | 13 | *Caenis* | GC | S2 | 170 | 0.02469 | 0.02128 | 0.00000 |
| Stream | 14 | *Cardiocladius* | PR | S1 | 1 | 0.00002 | 0.08511 | 0.00056 |
| Stream | 15 | *Chaetocladius* | GC | S2 | 3 | 0.00006 | 0.10638 | 0.03258 |
| Stream | 16 | *Cheumatopsyche* | FC | S7 | 424 | 0.07039 | 0.01064 | 0.00000 |
| Stream | 17 | *Chironomus* | GC | S1 | 60 | 0.00125 | 0.05319 | 0.02602 |
| Stream | 18 | Chrysomelidae | SH | S3 | 5 | 0.00010 | 0.26596 | 0.01267 |
| Stream | 19 | *Cinygma* | SC | S2 | 34 | 0.00141 | 0.12766 | 0.18574 |
| Stream | 20 | *Corbicula* | FC | S5 | 6 | 0.00025 | 0.08511 | 0.00411 |
| Stream | 21 | *Cricotopus* | GC | S0 | 62 | 0.00515 | 0.00000 | 0.00000 |
| Stream | 22 | *Crocothemis* | PR | S1 | 1 | 0.00002 | 0.08511 | 0.00056 |
| Stream | 23 | *Cryptochironomus* | PR | S1 | 1 | 0.00002 | 0.08511 | 0.00056 |
| Stream | 24 | *Culicoides* | PR | S4 | 2 | 0.00004 | 0.03191 | 0.09636 |
| Stream | 25 | *Demicryptochironomus* | GC | S2 | 2 | 0.00004 | 0.10638 | 0.03258 |
| Stream | 26 | *Dicranota* | PR | S4 | 1 | 0.00002 | 0.02128 | 0.00000 |
| Stream | 27 | *Dugesia* | OM | S6 | 53 | 0.00550 | 0.03191 | 0.00000 |
| Stream | 28 | *Eobrachycentrus* | SH | S1 | 4 | 0.00008 | 0.08511 | 0.00056 |
| Stream | 29 | *Ephemera* | GC | S3 | 35 | 0.00218 | 0.21277 | 0.07344 |
| Stream | 30 | *Ephemerella* | GC | S2 | 9 | 0.00019 | 0.10638 | 0.03258 |
| Stream | 31 | *Epoicocladius* | GC | S3 | 10 | 0.00021 | 0.26596 | 0.01267 |
| Stream | 32 | *Galba* | SC | S5 | 112 | 0.00465 | 0.08511 | 0.05444 |
| Stream | 33 | *Glossiphonia* | PR | S1 | 6 | 0.00025 | 0.11702 | 0.09609 |
| Stream | 34 | *Goerita* | SC | S3 | 2 | 0.00004 | 0.26596 | 0.01267 |
| Stream | 35 | *Gyraulus* | SC | S8 | 6 | 0.00012 | 0.03191 | 0.00000 |
| Stream | 36 | *Heptagenia* | SC | S0 | 46 | 0.00477 | 0.00000 | 0.00000 |
| Stream | 37 | *Hexatoma* | PR | S3 | 7 | 0.00015 | 0.26596 | 0.01267 |
| Stream | 38 | *Himalopsyche* | PR | S3 | 3 | 0.00006 | 0.26596 | 0.01267 |
| Stream | 39 | *Hippeutis* | SC | S5 | 45 | 0.00093 | 0.04255 | 0.02815 |
| Stream | 40 | *Hirudo* | PR | S6 | 9 | 0.00093 | 0.07447 | 0.06337 |
| Stream | 41 | Hydraenidae | PR | S5 | 1 | 0.00002 | 0.09574 | 0.01496 |
| Stream | 42 | *Hydrobaenus* | SC | S8 | 45 | 0.00093 | 0.03191 | 0.00000 |
| Stream | 43 | *Hydropsyche* | FC | S7 | 337 | 0.08392 | 0.01064 | 0.00000 |
| Stream | 44 | *Ilybius* | PR | S1 | 36 | 0.00075 | 0.05319 | 0.02602 |
| Stream | 45 | *Laccornellus* | PR | S2 | 2 | 0.00004 | 0.10638 | 0.03258 |
| Stream | 46 | *Larsia* | PR | S1 | 4 | 0.00008 | 0.05319 | 0.02602 |
| Stream | 47 | *Lebertia* | PR | S0 | 106 | 0.01980 | 0.00000 | 0.00000 |
| Stream | 48 | *Lepidostoma* | SH | S3 | 19 | 0.00158 | 0.03191 | 0.00000 |
| Stream | 49 | *Limnodrilus* | GC | S6 | 23 | 0.00286 | 0.04255 | 0.09975 |
| Stream | 50 | *Limnophora* | PR | S8 | 8 | 0.00033 | 0.03191 | 0.00000 |
| Stream | 51 | *Limnophyes* | GC | S4 | 5 | 0.00021 | 0.07447 | 0.16266 |
| Stream | 52 | *Lumbriculus* | GC | S5 | 13 | 0.00027 | 0.09574 | 0.01496 |
| Stream | 53 | *Macromia* | PR | S3 | 4 | 0.00017 | 0.26596 | 0.04759 |
| Stream | 54 | *Microtendipes* | FC | S2 | 2 | 0.00004 | 0.10638 | 0.03258 |
| Stream | 55 | *Natarsia* | PR | S9 | 204 | 0.04656 | 0.02128 | 0.01990 |
| Stream | 56 | Nematoda | PA | S2 | 5 | 0.00031 | 0.01064 | 0.00000 |
| Stream | 57 | *Nemoura* | SH | S3 | 4 | 0.00008 | 0.26596 | 0.01267 |
| Stream | 58 | *Neochauliodes* | PR | S4 | 2 | 0.00008 | 0.06383 | 0.09104 |
| Stream | 59 | *Notacanthurus* | SC | S3 | 1 | 0.00002 | 0.26596 | 0.01267 |
| Stream | 60 | *Ochrotrichia* | GC | S3 | 1 | 0.00002 | 0.26596 | 0.01267 |
| Stream | 61 | *Odontomyia* | GC | S4 | 1 | 0.00002 | 0.06383 | 0.06656 |
| Stream | 62 | *Oncomelania* | SC | S4 | 4 | 0.00008 | 0.07447 | 0.02328 |
| Stream | 63 | *Ordobrevia* | SC | S3 | 3 | 0.00006 | 0.26596 | 0.01267 |
| Stream | 64 | *Orectochilius* | PR | S3 | 10 | 0.00021 | 0.26596 | 0.01267 |
| Stream | 65 | *Ormosia* | GC | S5 | 2 | 0.00004 | 0.09574 | 0.01496 |
| Stream | 66 | *Orthocladius* | GC | S4 | 68 | 0.00847 | 0.02128 | 0.00000 |
| Stream | 67 | *Parachironomus* | PR | S4 | 4 | 0.00008 | 0.07447 | 0.02328 |
| Stream | 68 | *Paracricotopus* | GC | S4 | 12 | 0.00050 | 0.04255 | 0.00000 |
| Stream | 69 | *Parametriocnemus* | GC | S2 | 2 | 0.00008 | 0.12766 | 0.01418 |
| Stream | 70 | *Paraphaenocladius* | GC | S4 | 2 | 0.00004 | 0.02128 | 0.00000 |
| Stream | 71 | *Paratanytarsus* | GC | S8 | 3 | 0.00006 | 0.03191 | 0.00000 |
| Stream | 72 | *Paratendipes* | GC | S3 | 2 | 0.00004 | 0.26596 | 0.01267 |
| Stream | 73 | *Pedicia* | PR | S4 | 4 | 0.00008 | 0.07447 | 0.02328 |
| Stream | 74 | *Pericoma* | GC | S2 | 1 | 0.00002 | 0.10638 | 0.03258 |
| Stream | 75 | *Philoganga* | PR | S3 | 6 | 0.00012 | 0.26596 | 0.01267 |
| Stream | 76 | *Physa* | SC | S6 | 11 | 0.00068 | 0.04255 | 0.00435 |
| Stream | 77 | *Pila* | SC | S5 | 3 | 0.00006 | 0.09574 | 0.01496 |
| Stream | 78 | *Platysmittia* | GC | S5 | 7 | 0.00044 | 0.07447 | 0.00938 |
| Stream | 79 | *Polycanthagina* | PR | S3 | 18 | 0.00037 | 0.26596 | 0.01267 |
| Stream | 80 | *Polypedilum* | SH | S9 | 171 | 0.03903 | 0.01064 | 0.00000 |
| Stream | 81 | *Potamyia* | FC | S3 | 1 | 0.00002 | 0.01064 | 0.00000 |
| Stream | 82 | *Potthastia* | OM | S4 | 160 | 0.01328 | 0.02128 | 0.00000 |
| Stream | 83 | *Prosimulium* | FC | S1 | 3 | 0.00006 | 0.08511 | 0.00056 |
| Stream | 84 | *Pseudamophilus* | SC | S1 | 9 | 0.00019 | 0.08511 | 0.00056 |
| Stream | 85 | *Radix* | SC | S4 | 5 | 0.00021 | 0.06383 | 0.03935 |
| Stream | 86 | *Rheocricotopus* | GC | S2 | 5 | 0.00021 | 0.09574 | 0.04877 |
| Stream | 87 | *Rheotanytarsus* | FC | S4 | 8 | 0.00017 | 0.07447 | 0.02328 |
| Stream | 88 | *Rhyacophila* | PR | S3 | 14 | 0.00029 | 0.26596 | 0.01267 |
| Stream | 89 | *Serratella* | GC | S1 | 1 | 0.00002 | 0.08511 | 0.00056 |
| Stream | 90 | *Simulium* | FC | S6 | 195 | 0.02428 | 0.05319 | 0.06337 |
| Stream | 91 | *Smicridea* | FC | S5 | 2 | 0.00004 | 0.09574 | 0.01496 |
| Stream | 92 | *Stegopterna* | FC | S3 | 1 | 0.00002 | 0.26596 | 0.01267 |
| Stream | 93 | *Stictochironomus* | OM | S3 | 7 | 0.00029 | 0.25532 | 0.20407 |
| Stream | 94 | *Tabanus* | PR | S4 | 8 | 0.00050 | 0.04255 | 0.00000 |
| Stream | 95 | *Tanytarsus* | FC | S3 | 6 | 0.00025 | 0.21277 | 0.03365 |
| Stream | 96 | *Tipula* | SH | S6 | 33 | 0.00411 | 0.01064 | 0.00000 |
| Stream | 97 | *Trigomphus* | PR | S3 | 21 | 0.00087 | 0.29787 | 0.30290 |
| Stream | 98 | *Tubifex* | GC | S1 | 7 | 0.00044 | 0.08511 | 0.03569 |
| Stream | 99 | *Ugandatrichia* | SC | S3 | 2 | 0.00008 | 0.24468 | 0.04393 |
| Stream | 100 | *Zaitzevia* | GC | S3 | 4 | 0.00025 | 0.23404 | 0.00000 |
